# Multifaceted roles for BCL3 in cancer: a proto-oncogene comes of age

**DOI:** 10.1186/s12943-023-01922-8

**Published:** 2024-01-09

**Authors:** Gillian Seaton, Hannah Smith, Andrea Brancale, Andrew D. Westwell, Richard Clarkson

**Affiliations:** 1 European Cancer Stem Cell Research Institute, Cardiff University School of Biosciences, Hadyn Ellis Building, Maindy Road, Cardiff, CF24 4HQ UK; 2 UCT Prague, Technická 5, 166 28, 6 – Dejvice, IČO: 60461337 Prague, Czech Republic; 3 Cardiff University School of Pharmacy and Pharmaceutical Sciences, Redwood Building, King Edward VII Avenue, Cardiff, CF10 3NB UK

**Keywords:** BCL3, Transcription, Hallmarks, WNT, NF-kappaB, STAT3, SMAD3, C-Myc, Targeted therapy

## Abstract

In the early 1990’s a group of unrelated genes were identified from the sites of recurring translocations in B-cell lymphomas. Despite sharing the nomenclature ‘Bcl’, and an association with blood-borne cancer, these genes have unrelated functions. Of these genes, BCL2 is best known as a key cancer target involved in the regulation of caspases and other cell viability mechanisms. BCL3 on the other hand was originally identified as a non-canonical regulator of NF-kB transcription factor pathways – a signaling mechanism associated with important cell outcomes including many of the hallmarks of cancer. Most of the early investigations into BCL3 function have since focused on its role in NF-kB mediated cell proliferation, inflammation/immunity and cancer. However, recent evidence is coming to light that this protein directly interacts with and modulates a number of other signaling pathways including DNA damage repair, WNT/β-catenin, AKT, TGFβ/SMAD3 and STAT3 – all of which have key roles in cancer development, metastatic progression and treatment of solid tumours. Here we review the direct evidence demonstrating BCL3’s central role in a transcriptional network of signaling pathways that modulate cancer biology and treatment response in a range of solid tumour types and propose common mechanisms of action of BCL3 which may be exploited in the future to target its oncogenic effects for patient benefit.

## Background

 BCL3 was originally identified as the site of a recurring translocation at chromosome 19;q13.32 in patients with chronic lymphocytic leukemia (CLL) [1] and was subsequently shown to be associated with overexpression of the BCL3 gene in a range of B-cell malignancies [1–3]. The nomenclature ‘Bcl-33’ is often used interchangeably in the literature for this proto-oncogene, but strictly the BCL3 gene locus was identified a year previously as the site of a c-Myc translocation rearrangement on chromosome 17q:22 in a patient with a Bcl2 associated aggressive prolymphocytic leukemia [4] also identified in a number of follicular lymphomas [5]. Thus, for clarity and consistency with the family of genes identified in CLL, alongside the apoptosis regulator BCL2, we propose that the nomenclature BCL3 is most appropriate for this proto-oncogene.

**Fig. 1:**
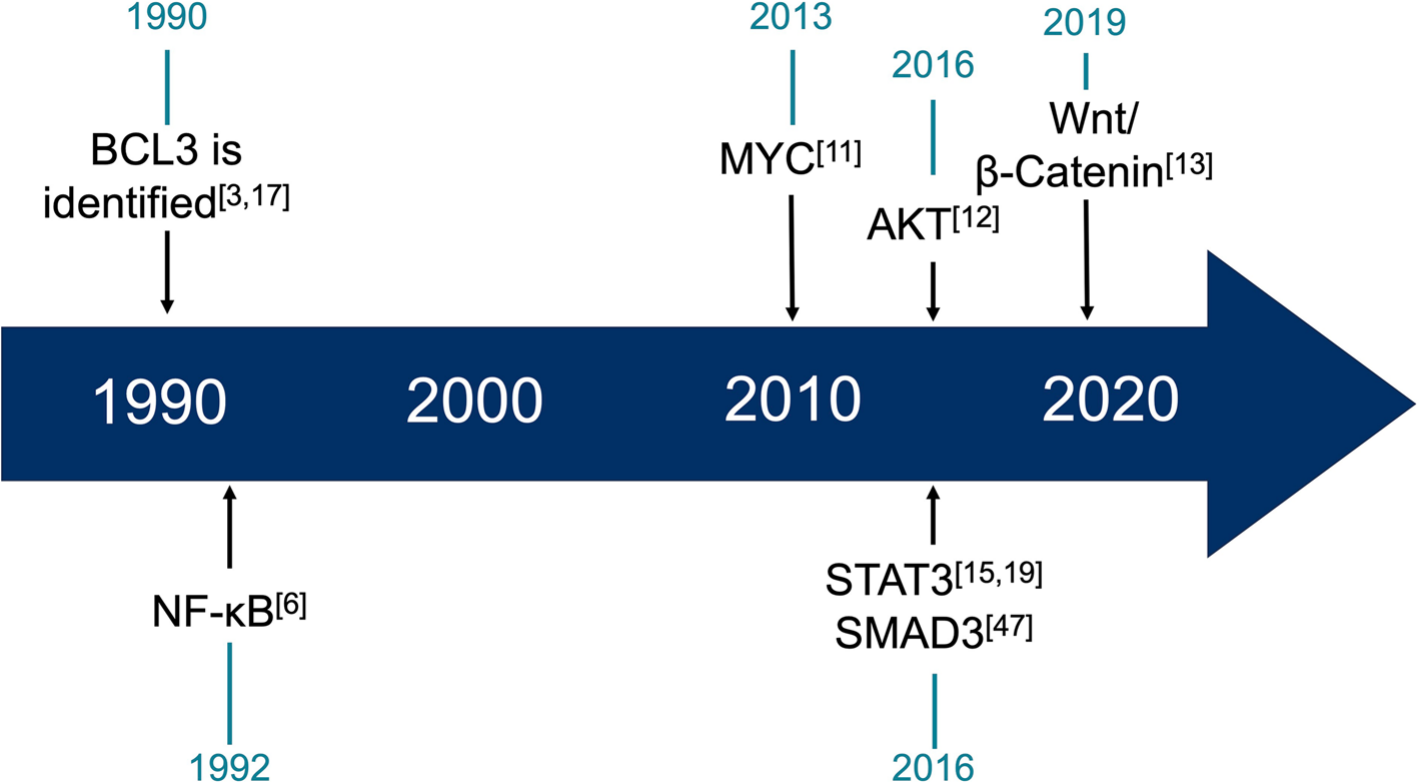
Discovery timeline of BCL3 and its role in cancer-related signaling. Since its initial identification and early discovery as an NF-kB co-factor, BCL3 has only relatively recently been shown to directly modulate additional transcription factors and cell-signaling pathways associated with cancer hallmarks

**Fig. 2:**
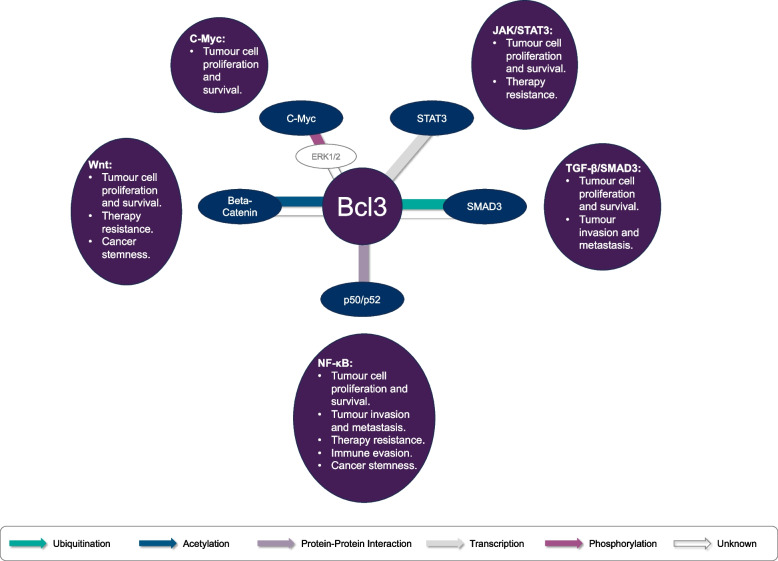
BCL3: a master modulator of gene transcription networks. BCL3 has been reported to directly impact on five transcription factor pathways (NF-kB, WNT/β-cateinin, c-Myc, JAK/STAT3 and SMAD3) in cancer cells. The mechanism by which BCL3 mediates its effects varies for each pathway and depends on the cancer cell context. This includes BCL3’s direct protein:protein interaction with, or indirect protein modification (acetylation, phosphorylation or ubiquitination) of, the target transcription factors or their co-regulators; or via binding of nuclear BCL3 complexes to the transcription factor gene promoter. The outcome of BCL3’s modulation of these transcription factors on cancer cell phenotypes depends on the transcription factor pathways themselves, as indicated. We have restricted this summary to mode of transcription factor regulation directly illustrated in cancer cells and further information on the BCL3 interactome in other contexts can be obtained in [16]

Sequence analysis of the BCL3 locus identified a transcript containing a seven tandem (Ankyrin) repeat motif present in yeast cell cycle control genes [3] and homology to the IkB family of NF-kB transcription factor regulators [6]. Subsequent elucidation of BCL3’s role in regulating NF-kB subunits [6–9] accompanied a period of intense research activity around the regulation and function of NF-kB in normal and pathological contexts including structural characterization of the BCL3 ANKYRIN repeat motif essential for its function [10]. Perhaps not unsurprisingly, it wasn’t until two decades later that alternative mechanisms of action of BCL3, involving direct interaction and regulation of NFkB-independent signaling proteins were identified. These alternative pathways included c-Myc, WNT, AKT, STAT3 and SMAD3 [11–15]; all of which have important roles in tumourigenesis and cancer progression (Fig. 1). These more recent studies confirm that BCL3 plays a central role in modulating a network of cancer related transcription factors (Fig. 2) and other key oncogenic signals that enable hallmarks of cancer [16] and modulate cancer progression [17].

In this review we will focus on the evidence that demonstrate the direct effect of BCL3 on these oncogenic pathways, highlighting commonality in the mechanism underpinning their regulation and demonstrating the cancer modifying role that BCL3 plays in a variety of solid tumour types. We have restricted our discussion to events occurring in cancer cells, for further information on BCL3 regulation, signaling and the BCL3 interactome in other contexts we refer you to previous excellent reviews ([16–18].

### BCL3 and NF-kB signaling

The NF-κB family represents a highly conserved group of transcription factors, that contribute towards context-dependent cellular responses to a variety of extracellular stimuli, such as tumour necrosis factor-alpha (TNF-α), receptor activator of nuclear factor kappa-B ligand (RANKL) and B-cell activating factor (BAFF). The dimeric transcription factors mediate a multitude of biological processes associated with the hallmarks of cancer; including the regulation of the immune response, inflammation, apoptosis/cell survival, cell motility and cell proliferation. Consequently, aberrant NF-κB signaling is associated with pro-oncogenic mechanisms involving tumour initiation and progression to metastasis and therapy resistance [19, 20].

The mammalian NF-κB protein family consists of five structurally similar REL proteins (RelA, RelB, c-Rel, NFkB1 and NFkB2 genes), each possessing a REL homology domain (RHD) to allow the dimerisation of subunits and the interaction of the dimers with target sites on DNA [21]. Additionally, RelA, RelB and c-Rel also possess a transactivation domain, responsible for the transactivation of target genes; this domain is not present on NF-κB1 (p105) and NF-κB2 (p100), or the mature peptides produced post-modification (p50 and p52, respectively). As a result, the presence of different heterodimer and homodimer combinations within the NF-κB signaling cascade can result in an extensive variation in target gene expression, contributing towards the context-dependency of the pathway. As p50 and p52 homodimers lack transactivation domains, they cannot induce transcription of target genes alone, and require the presence of BCL3 to initiate gene transcription [22].

BCL3 mediates its modulatory role on NF-kB signals through direct protein-protein interactions with the subunits p50 and p52 via its structurally well-defined ANKYRIN repeat [23, 24]. Predicting the consequences of this complex formation on downstream transcriptional target genes can be challenging however, as both gene activation and gene suppression roles have been attributed to BCL3 in different studies involving different experimental models [25, 26]. The more widely reported BCL3 responses involve transactivation, whereby BCL3 binding either provides the transactivating domain required to recruit transcription co-factors and drive target gene transcription [22–24, 27], or sequesters the inhibitory subunits from gene promoters allowing transactivating dimers to bind in their place [28]. In contrast, BCL3 mediated downregulation of NF-kB responsive genes occurs either through stabilisation of inhibitory subunits or recruitment of HDACs at gene promoters [29, 30]. Which mechanisms predominate in a particular context is unclear, but likely depends on both the target promoter sequence and additional post-translational regulation of this highly phosphorylated and ubiquitinated protein [31–33]. It is not uncommon for both upregulated and downregulated genes to be identified in the same experimental model when BCL3 activity is altered. For example, p52/BCL3 complexes upregulate G/C centric promoter sites via direct recruitment of the acetyltransferase Tip60 while A/C centric promoters are downregulated in the same cells through HDAC3 recruitment [32]. Notwithstanding these differences, the ability of BCL3 to bind to its cognate NF-kB protein partners via its ANKYRIN domain is a pre-requisite for its further post-translational modification and ability to modulate NF-kB signaling [33].

As both p50 and p52 subunits are direct interactive partners of BCL3, both canonical and non-canonical NF-kB pathways are modified by BCL3 regulation, yet complete loss of BCL3 activity does not abrogate all NF-kB signaling. This is best illustrated in BCL3 knockout mice which, apart from deficiencies in humoral immune responses, are viable [34, 35] which contrasts with more severe phenotypes observed in knockouts of the NF-kB transcription factor subunits [36].

In summary, BCL3 provides for the subtle modulation of NF-kB pathways through direct protein interactions with a subset of NF-kB subunits leading to both up-regulation and down-regulation of target genes.

### BCL3 and c-Myc

The first alternative target of BCL3 that was independent of NF-kB signaling to be identified was the proliferative transcription factor c-Myc. C-Myc is a canonical proliferation-related transcription factor that plays a significant role in cell cycle progression, apoptosis, and cellular transformation by directly controlling gene expression through binding to E-box motifs in cell cycle related target gene promoters including (but not restricted to) cdc2, Cdc25A, cyclinD1 and cyclin A. C-Myc regulates transcription through several mechanisms, and is estimated to target as many as 15% of all genes [37]. C-Myc protein levels are tightly regulated, with a half-life of about 20–30 min due to quick turnover through the ubiquitin–proteasome system [38]. GSK3-β and ERK1/2 differentially regulate c-Myc through phosphorylation events at Threonine 58 (Thr58) and at Serine 62 (Ser62), respectively, events that are required for the E3 ubiquitin ligase Fbw7 to regulate c-Myc stability [39]. In colorectal cancer cells BCL3 promotes phosphorylation of c-Myc via ERK1/2, extending the half-life of c-Myc and reducing levels of ubiquitinated protein ensuring c-Myc prolonged presence and consequently its sustained transcriptional activity. Thus, the combined overexpression of BCL3 and c-Myc in certain cancers may lead to a synergistic oncogenic effect. By contrast, the same authors showed no change in AKT/GSK3-β signaling in response to BCL3 manipulation, suggesting the c-Myc effects were specific to the Serine 62 phosphorylation site [11].

Alternatively, BCL3 may transcriptionally regulate c-Myc gene expression. This has been reported in MDA-MB-468 triple negative breast cancer cells following BCL3-shRNA mediated suppression but the underlying transcriptional mechanism and c-Myc promoter binding sites responsible for this regulation have not been elucidated [40]. Strategies that inhibit BCL3 therefore could lead to reduced c-Myc expression via one or more mechanisms, potentially providing a therapeutic effect in cancers where this interaction is pivotal.

### BCL3 and the JAK/STAT3 axis

The JAK/STAT pathway is a canonical signaling cascade triggered by a range of cytokines and growth factors. When ligands bind to their respective receptors, they cause receptor dimerization, activating associated JAKs. These JAKs then phosphorylate the receptor, providing docking sites for STAT proteins. Once bound, STATs are phosphorylated by JAKs, leading them to dimerize, translocate to the nucleus, and initiate transcription of target genes. STAT3 is one of the seven mammalian STAT proteins and plays a pivotal role in mediating responses to many cytokines and growth factors. STAT3 shares a similar relationship with cancer as NF-kB, being stimulated by similar cancer-related pro-inflammatory signals and impacting a number of overlapping cancer hallmarks including proliferation, migration and cancer immunity [41]. BCL3 has been recognized for some time to be a direct transcriptional target of STAT3 in a cancer context [42–44] yet the reciprocal relationship was not described until relatively recently [15, 45, 46].

In Multiple Myeloma (MM), but not hepatocellular carcinoma, ChIP and Luciferase gene reporter assays demonstrated IL-6 to regulate BCL3 expression via the binding of STAT3 to the HS4 BCL3 gene enhancer. Knockdown of STAT3 via siRNA decreased BCL3 protein expression in MM cell lines. The authors also demonstrated that BCL3 can negatively regulate its own transcription, via simultaneous binding with p50 to the HS3 gene enhancer, which could be attributed to maintaining basal cellular levels of BCL3 [42].

BCL3 has been shown to enhance STAT3 transcription in certain contexts. BCL3 regulation of STAT3 expression has been described in cervical [45], and glioblastoma cell lines [15], where shRNA knockdown of BCL3 resulted in decreased STAT3 and in the latter, pSTAT3 protein levels, while overexpression of BCL3 promoted the increase in STAT3 protein levels in both cell types. However, no studies to date have identified precisely how BCL3 regulates STAT3 protein levels. Due to the reciprocal nature of this BCL3/STAT3 axis, in cancers where both BCL3 and JAK/STAT3 signaling are upregulated, combination therapies targeting both might be beneficial.

### BCL3 and SMAD3

SMAD3 is a member of the SMAD family of proteins, which are central to the transforming growth factor-beta (TGF-β) signaling pathway. TGF-β signaling is known to have dual roles in cancer: it can suppress tumor formation in early stages, but promote tumor progression and metastasis in established tumors. BCL3 is implicated in transforming growth factor-β (TGF-β) signaling through its direct interaction with the transcription factor SMAD3. TGF-β binds to transmembrane receptors, initiating phosphorylation and translocation of SMAD3 complexes to the nucleus, where they bind to DNA to regulate target genes [47]. shRNA knockdown of BCL3 in breast cancer cells with concomitant TGF-β stimulation, revealed decreased SMAD3 phosphorylation, decreased SMAD3 protein levels and attenuated TGF-β reporter luciferase assay activity [14]. SMAD3 protein levels were rescued in BCL3 knockdown cells by proteasome inhibitor MG132 treatment, implicating BCL3 in SMAD3 degradation. The authors showed co-localisation of SMAD3 and BCL3 in the nucleus upon TGF-β stimulation, and a direct interaction between the MH2 domain of SMAD3 and BCL3 via co-immunoprecipitation, suggesting that protein binding sequesters SMAD3 from proteasomal degradation, independent of phosphorylation signals. Interestingly, the rescue experiment, of depleting the E3 ubiquitin ligases that bind the MH2 domain of SMAD3 failed to restore SMAD3 protein levels in BCL3 knockdown cells, suggesting some alternative mechanism of BCL3-mediated stabilisation. Additional evidence for binding of BCL3 to stabilise SMAD3 is limited to a study of vascular smooth muscle cells whereby suppression of BCL3 was shown to enhance apoptosis and suppress proliferation in a model of abdominal aortic aneurisms [48]. It is worth noting however that a reciprocal relationship may also exist in some cancer cell types, as p-SMAD3 has been shown to bind to the BCL3 promoter, upregulating BCL3 in response to TGF-β [49].

### BCL3 and AKT signaling

The AKT pathway is a critical regulator of various cellular processes, including cell proliferation, survival, growth, and metabolism. Activation of AKT signaling often begins with growth factors binding to receptor tyrosine kinases (RTKs), leading to the activation of phosphoinositide 3-kinase (PI3K). PI3K then produces phosphatidylinositol-3,4,5-triphosphate (PIP3), facilitating AKT activation. In cancer, the PI3K/AKT pathway is one of the most frequently over-activated intracellular pathways, acting on different downstream target proteins, to contribute to proliferation, invasion, and metastasis of tumour cells [50]. A number of studies have investigated a link between BCL3 and AKT. In colorectal carcinoma cells overexpression of BCL3 promoted both Ser473 and Thr308 phosphorylation of AKT and downstream target FOXO1/3a and GSK3β [12], while siRNA knockdown of BCL3 decreased phospho-AKT and downstream targets, in response to hypoxia. AKT Ser473 phosphorylation did not occur on overexpression of an NF-kB binding mutant version of BCL3 suggesting that AKT phosphorylation was dependent on the BCL3:NF-kB axis. This relationship was supported by a study of ApoE-deficient psoriatic mice that demonstrated protein expression of phospho-AKT (p-AKT) and p-GSK3β was decreased by silencing of BCL3 [51].

There is also evidence of reciprocity observed in macrophages whereby AKT directly phosphorylates Ser33 on BCL3 and GSK3-β phosphorylates Ser394 and Ser398 leading to poly-ubiquitination on Lysine residues K13 and K26 of BCL3 to promote its nuclear localization and degradation respectively [29, 52]. It remains to be determined if this reciprocal relationship exists in cancer cells, but it seems likely from the evidence to date that aberrant AKT signaling in cancer cells is impacted both by and on BCL3 activity.

### BCL3 and WNT signaling

The precise regulation of the WNT signaling cascade promotes the development and maintenance of healthy tissue, through the mediation of cellular processes including cell proliferation, cell migration and apoptosis [53]. Hence, aberrant WNT signaling is widely associated with tumorigenesis and maintenance, and contributes towards the tumour-specific treatment response and stem-like properties within the tumour population associated with a plethora of malignant conditions [54].

Recent evidence demonstrates that BCL3 can modulate β-catenin/TCF-mediated transcription. Complex immunoprecipitation (Co-IP) studies within mouse embryonic stem cells (mESCs) ([55] and later in CRC cell lines [13, 56]) revealed a direct interaction between BCL3 and β-catenin; various BCL3 mutant constructs were used to identify a region immediately 5′ of the ANK-repeat region of BCL3 (between amino acids 31 and 125) which were required for β-catenin complex formation [56]. β-Catenin/TCF reporter activity was demonstrated to correlate with overexpression or targeted suppression of BCL3, and was dependent on WNT pathway mutation status [13]. The effect of BCL3 suppression on a selection of canonical and stem-cell specific WNT target genes demonstrated the downregulation of LGR5 and ASCL2 expression, whereas the canonical WNT targets remained unaffected [13] suggesting that BCL3 acts as a context dependent modulator of β-catenin/TCF-mediated transcription, potentially restricted to stem-like tumour cells. BCL3 suppression also impeded stem-like properties both in vitro [13] and in vivo [56], further validating this theory.

Additionally, BCL3 is demonstrated to promote WNT activity by influencing the post-translational modification of β-catenin. BCL3 suppression in both HCT116 and SW620 cell lines resulted in a decrease in ac-K49 β-catenin protein expression, as determined by western blot and immunofluorescence (IF) analysis [56]. This decrease was rescued via the addition of HDAC inhibitors, and accompanied by an increase in β-catenin/TCF reporter activity. Further investigation via Co-IP studies, demonstrated an increased interaction of β-catenin and HDAC-1, but not HDAC-6, within BCL3 suppressed HCT116 cells; an increase in HDAC-1 protein expression was also observed [56]. It was therefore concluded that BCL3 mediates both HDAC-1 protein expression, as well as interrupting the interaction of HDAC-1 with β-catenin, resulting in aberrant acetylation of β-catenin and the consequent activation of β-catenin/TCF-mediated transcription, within CRC cells.

Indirect effects of BCL3 on mechanisms impacting on WNT/APC signaling pathways have also been reported. One study identified a novel regulator of WNT-mediated transcription, CtBP1 [57], that was stabilised when bound to the carboxy terminal region of BCL3, resulting in the suppression of apoptosis [58]. BCL3 has also been suggested to be a key determinant in the COX-2-mediated response to inflammatory cytokines in colorectal tumour cells, promoting PGE2-driven tumorigenesis [59] – a pathway known to be involved in several hallmarks of cancer and associated with WNT signaling [60].

WNT-mediated regulation of BCL3 has also been described, however the relationship appears to be complex. Expression of BCL3 and β-catenin was inversely correlated in colorectal cancer cell lines and targeted knockdown of β-catenin promoted BCL3 expression [13], while stimulation of the pathway by exogenous Wnt3a administration also led to an increase in BCL3 expression in the same cell lines [56]. Further studies are required to understand the mechanisms underpinning the control of BCL3 expression by the WNT pathway.

## BCL3 and Cancer

BCL3 is an established oncogene in hematologic malignancies, having been originally identified at a recurring translocation site in patients with B-cell chronic lymphoma and subsequently identified to be elevated in patients with anaplastic large cell lymphoma. The t(14;19)(q32;q13) is a balanced translocation found in less than 1% of lymphoid neoplasms where Bcl3 is involved in regulation of cell proliferation, differentiation, and survival of B and T-cells. Bcl-3 mutations in lymphocytes give rise to overexpression of IκB protein and uncontrolled cell proliferation, which lead to B-cell chronic lymphocytic leukemia [1, 61]. The t(14;19) and BCL3-rearrangement (BCL3-R) have been identified in a broad spectrum of different tumor subtypes [62–66] and whilst BCL3 was originally identified in CLL, in a cytogenetic analysis of 4487 tumors diagnosed as lymphoproliferative disorder, the t14;19)q32;q13 was found in six cases [2], suggesting the translocation is quite rare. t(14;19) and BCL3-R have been identified in diffuse large B-cell lymphomas (DLBCL), marginal zone lymphomas (MZL), splenic small B-cell lymphomas, and tumors diagnosed as B-cell non-Hodgkin lymphomas [65, 66]. A study by Carbó-Meix and colleagues in 2023 sought to clarify the genomic configuration of BCL3-R in B-cell neoplasms via integrative whole-genome sequence, transcriptomic, and DNA methylation analysis of 13 lymphoid neoplasms and correlate the biological significance with patient clinical and pathological characteristics. The authors found upstream or downstream breakpoints of BCL3-R are mainly associated with two subtypes of lymphoid neoplasms with different (epi)genomic, expression, and clinicopathological features resembling atypical CLL and MZL, respectively [67]. BCL3 expression has an important function in classical Hodgkins Lymphoma (cHL) and peripheral T-cell non-Hodgkin lymphoma (T-NHL), in particular ALCL [68].

**Fig. 3:**
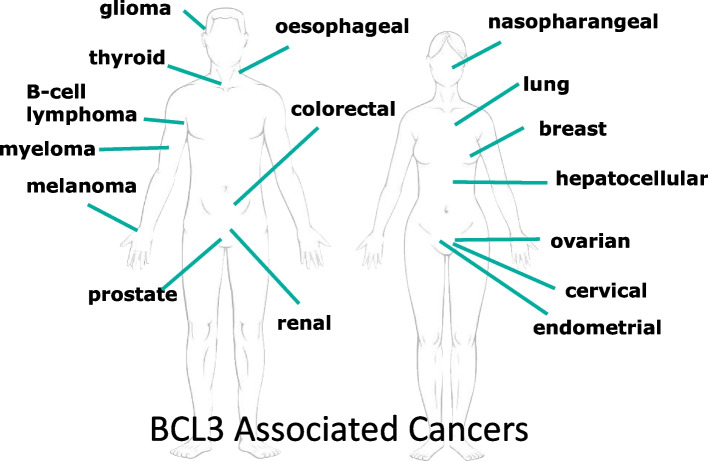
Cancer tissue types associated with elevated BCL3. Graphical representation of the studies listed in Table 1, showing the tissue-of-origin tumours where BCL3 has been shown to be aberrantly expressed and/or linked to poor outcome

Since its initial identification in 1990, it wasn’t until the early 2000’s that BCL3’s potential role in solid tumours was recognized (Table 1). BCL3 is now known to be upregulated in solid tumours from at least 16 different tissue origins (Fig. 3), of which 14 are solid tumour sites, and has been shown to be an independent prognostic indicator of overall survival or metastatic free survival in a large proportion of these, suggesting a key role as a driver in disease progression (Table 1).

**Table 1:** BCL3 is linked to multiple tumour types. A summary of the publications demonstrating that BCL3 expression is elevated in tumour versus normal tissue and/or correlation between BCL3 expression and poor prognosis (overall survival or metastasis free survival), listed by tumour type

Tumour type	Elevated expression cancer vs normal	Prognostic Biomarker
Nasopharyngeal/oral	Andrews, 2000 [69] Thornburg, 2003 [70] Mishra, 2006 [71] Chung, 2013 [72]	
Non-Hodgkin Lymphomas	Canoz, 2004 [62]	
Hodgkin Lymphomas	Rassidakis, 2003 [73] Canoz, 2004 [62]	
AML		Niu, 2019 [74]
Myeloma	Brenne, 2009 [75]	Brenne, 2009 [75]
Breast	Pratt, 2003 [76] Huo, 2018 [40] Chen, 2016 [14] Schulten, 2017 [77]	Huo, 2018 [40] Chen 2016 [14] Czapiewski, 2022 [78]
Endometrial	Pallares, 2004 [79]	
Hepatocellular	O’Neil, 2007 [80] Tu, 2016 [81]	Tu, 2016 [81]
Melanoma	Uffort, 2009 [82]	Diaz-Ramon, 2023 [83]
Ovarian	Zou, 2018 [84]	
Prostate	Ahlqvist, 2013 [85]	
Renal cell carcinoma	de Souza Braga, 2014 [86] Dai, 2016 [87]	
Colorectal	Saamarthy 2015 [88] Tao, 2018 [89]	Tao, 2018 [89] Zhang, 2020 [90]
Cervical	Zhao, 2016 [45]	Zhao 2016 [45]
Gioblastoma	Wu,2016 [15] Fan, 2022 [91]	Wu, 2016 [15] Wu, 2018 [92] Fan, 2022 [91]
Oesophageal	Puccio, 2018 [93] Soares-Lima, 2021 [94]	
SC- Lung carcinoma	Dimitrakopoulos, 2015 [95]	
NSC – lung carcinoma		Dimitrakopoulos, 2019 [96]
Thyroid		Xiao, 2022 [97]

Despite these numerous associations with poor prognosis, the demonstration of a direct role for BCL3 as an oncogenic driver or disease modifier has only been demonstrated by experimentation in a handful of cancer types to date. While it may only be a matter of time before causality is confirmed in the majority of other solid tumour types, for the purpose of this review we will restrict our discussion to the evidence demonstrating the pro- and anti-oncogenic effects associated with the experimental intervention (increase or decrease) of BCL3 activity distinguishing these from where correlative associations with BCL3 expression are implied.

Early studies of the oncogenic role of BCL3 naturally focused on NF-kB mediated pathways in cancer, most notably the transcriptional control of cyclin D1 and p53 to regulate cell cycle [98–103], and cell viability (including p53 mediated apoptosis) [58, 104–106]. More recently, as other regulatory mechanisms have come to light, it has become clear that BCL3 impacts several of the ‘acquired capabilities’ and ‘enabling characteristics’ outlined as the key Hallmarks of Cancer [107]. Thus, in additional to its role in tumour cell cycle and cell survival it plays a disease modifying role in solid tumours by promoting tumour cell invasion, cancer stemness and the tumour protective microenvironment; while also impacting on therapeutic resistance and DNA damage repair pathways. Below, we summarise the evidence demonstrating causative roles for BCL3 in each of these cancer related hallmarks.

### BCL3 and tumour cell proliferation and survival

Each of the five transcription factor pathways regulated by BCL3 identified to date (Fig. 2) are canonical cell cycle related pathways in cancer. Cell cycle related genes common to these pathways and shown to be regulated by BCL3 include (but are not restricted to) Cdc2, cyclinD1 and CyclinE. While there is direct evidence for BCL3 as a transactivator of cell cycle genes [102], the direct evidence for BCL3 mediated regulation of cell cycle genes in tumour cells has focussed on NF-kB mediated regulation of cyclinD1 in NSCLC, melanoma, hepatocellular carcinoma and osteocarcinoma cell lines [81, 99, 108]; CDK1 in glioblastoma [109]; p27 and c-Myc in triple negative breast cancer cells [40]; STAT3 mediated upregulation of cyclinD1 in glioma cells [15] and c-jun transcription factor (which regulates cyclinD1 transcription) in colorectal cell lines [56].

**Fig. 4:**
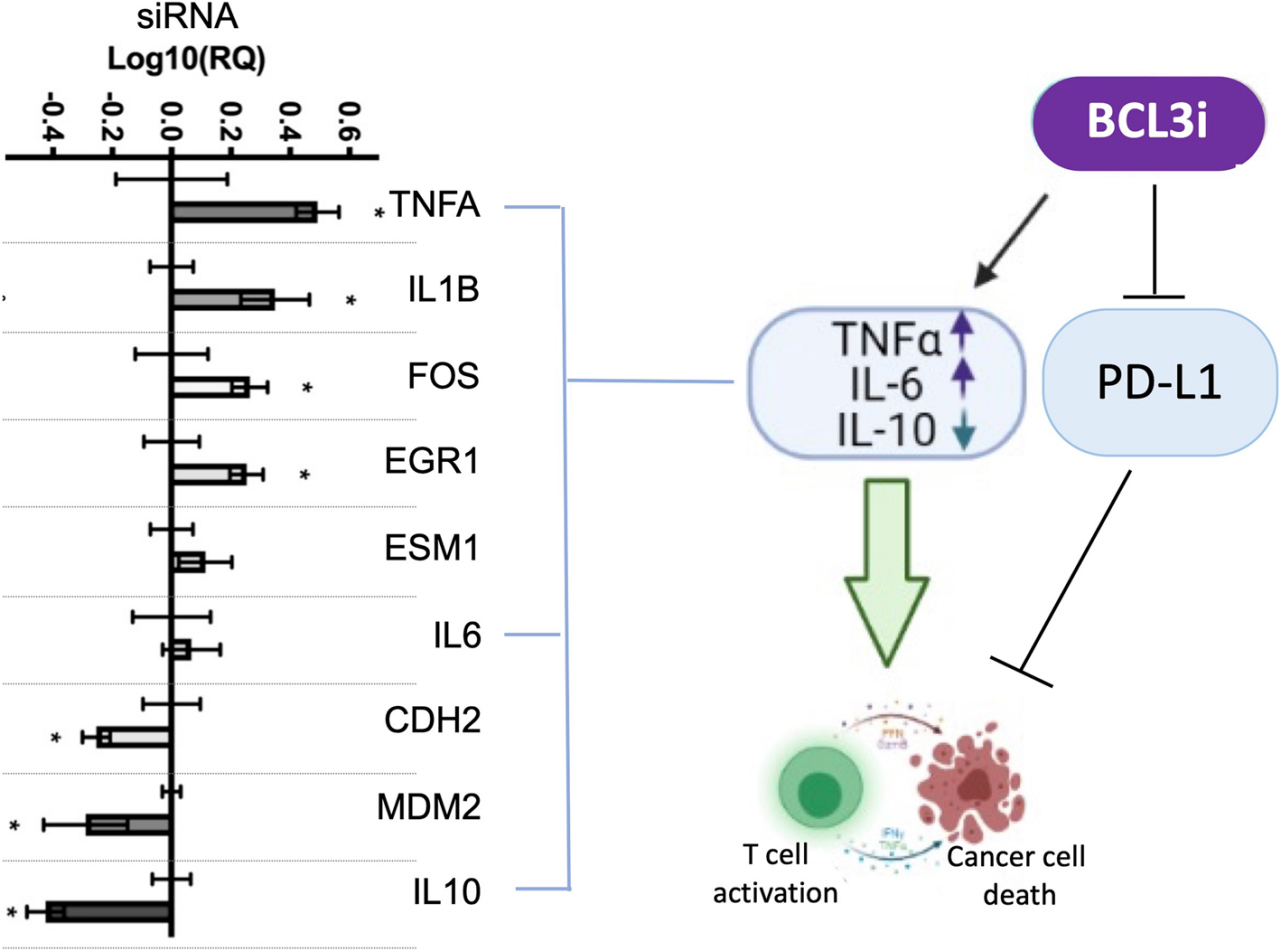
Transcription responses to BCL3 suppression predict alterations to the tumour microenvironment. BCL3 simultaneously promotes and represses different BCL3-responsive genes in a cell-context manner, as illustrated by the co-regulation of a subset of BCL3-responsive gene changes following BCL3 targeted siRNA in the breast cancer cell line MDA-MB-231 (left panel; adapted from [111]). These transcriptional signatures predict changes to the tumour microenvironment, for example that might impact on tumour surveillance, supported by independent observations on the effect of BCL3 on checkpoint-control (right panel [84, 127];). Created with BioRender.com

Other examples of a direct involvement of BCL3 on cell proliferation and tumour growth, but where the specific mechanism of cell cycle regulation was not defined, include a study of cervical cancer cell lines which demonstrated a STAT3 dependent regulation of G1/S progression and a reduction in xenograft tumour growth following shRNA targeting BCL3 [45].

Similarly, there are several studies demonstrating a direct role for BCL3 in promoting tumour cell survival, which impacts on tumour growth and viability, yet often the precise mechanism of action of BCL3 remains elusive. For example, suppression of BCL3 in HeLa cells subjected to UVB radiation exhibited increases in the expression of the apoptotic regulator, tBid along with elevated caspase cleavage and a decrease in the anti-apoptotic protein BCL2 [110]. BCL3 promoted tumour growth in mouse xenografts of colorectal cancer was directly linked to an AKT-mediated suppression of apoptosis which was dependent upon BCL3’s interaction with p50 or p52 homodimers [12]. These authors rescued the pro-apoptotic effect of siRNA-BCL3 by expressing constitutively active AKT in HCT116 cells yet the mechanistic link between the NF-kB complexes and the PI3K/AKT pathway was not determined. In contrast, the mechanism of action of BCL3 in controlling apoptosis in glioma cells was defined as a STAT3 mediated regulation of BCL2 and Mcl1 while suppression of BCL3 reduced xenograft tumour growth.

In both breast and prostate cancer, the current evidence points to BCL3 having more pronounced effects on cell survival mechanisms and promoting tumour progression (see metastasis section below) than cell cycle regulation. Thus, growth of ErbB2 mammary tumours exhibited a non-significant reduction in constitutive BCL3 knockout mice compared to controls expressing normal levels of BCL3, with no significant changes in cyclinD1 or caspase3 levels within the mammary tumours. Instead, a significant decrease in cell turnover was observed at the metastatic sites, suggesting that primary tumour growth was less affected by BCL3 loss than at distal lesions [15]. Pharmacological suppression of BCL3 in adult mice however led to a significant reduction in tumour volume in two xenograft models of breast cancer. This was accompanied by significant increases in cleaved caspase 3-positive cells but no significant difference in phospho-histone H3 within the tumours, suggesting that the primary effect of targeting BCL3 in breast tumours was an increase in apoptosis rather than a reduction in cell proliferation [111]. Similarly, enforced suppression of BCL3 by shRNA in DU145 prostate cancer xenografts resulted in a significant reduction in tumour growth which correlated with an increase in the number of cleaved caspase-3 positive cells within the treated tumours but no change in mitotic index [85]. Moreover the direct effect of BCL3 on the stability and protein levels of the apoptosis associated protein CtBP1 was demonstrated in the MCF7 breast cancer cell line and further supported by correlative expression in primary breast cancer tissues [58], implying an indirect effect on WNT/APC mediated survival pathways [57] in cell survival of breast cancer cells. These findings highlight the importance of considering the context of BCL3 intervention on the tumour phenotype. Thus, suppression of BCL3 in adult tissues (more akin to a clinical intervention context) appears to be more efficacious than when BCL3 is deleted from birth.

### BCL3 and tumour invasion/metastasis

In contrast to the modest effects of BCL3 on breast tumour growth kinetics, spontaneous metastasis of BCL3-null tumours was found to be dramatically reduced compared to their BCL3 controls. Metastatic ErbB2 (HER2) tumour burden was reduced by 75% in BCL3-null mice and this was recapitulated in allografts of BCL3-null tumour cells into wild-type recipient mice, confirming the tumour cell autonomous effects of BCL3 on the metastatic process [44]. This finding was subsequently confirmed in independent studies which demonstrated the potent anti-metastatic effects of inhibiting BCL3 by shRNA in human breast cancer xenografts [14] and a direct correlation between BCL3 expression and metastatic potential in vivo [112].

A direct role for BCL3 in tumour cell migration/invasion has also been demonstrated in melanoma and gastric carcinoma cell lines [108, 113] whereby siRNA mediated knockdown of BCL3 expression resulted in a reduction in cell migration in in vitro (scratch) assays of cell motility, which in melanoma cells was accompanied by the loss of N-cadherin [108], attributed to the recruitment of BCL3 to an NF-kB binding site in the N-cadherin promoter.

Direct regulation of N-cadherin might suggest a role for BCL3 in epithelial to mesenchymal transition (EMT), and this is supported by demonstration of BCL3’s effects in mesenchymal differentiation and the regulation of EMT-related genes in A172 glioma cells [92], yet the weight of evidence in other tumour cell types suggests that while BCL3 may be regulated during EMT-MET flux, its influence is limited to the regulation of a subset of EMT and migration related genes and may not of itself induce EMT [108, 112]. Notwithstanding its inability to drive EMT, BCL3’s impact as a permissive regulator of cell migration encompasses a spectrum of motility phenotypes whereby suppression of BCL3 results in the inhibition of both constitutive and EMT-acquired cell motility, restricting all forms of cancer cell motility in a non-redundant manner [112]. Mechanisms underpinning the effects of BCL3 on breast cancer cell migration include NF-kB/BCL3 mediated transcriptional regulation of N-cadherin and cdc42 [112] and protein stabilization of SMAD3 induced by TGF-β [14] while in a mesenchymal subset of glioblastoma this TGF-β / SMAD3 axis drives BCL3 transcription – suggesting that in some cancer contexts a positive feedback exists between BCL3 and SMAD3 to drive disease progression, mediated ultimately through upregulation of BCL3.

### BCL3 and genome integrity

Early indications of a role for BCL3 in genome integrity were posited when BCL3 was shown to suppress p53 via upregulation of Hdm2 in response to UVB induced DNA damage of breast cancer cells [105] and when shRNA suppression of BCL3 was shown to induce aneuploidy in HeLa cells with accompanying increases in phospho-ATM, gamma-H2Ax and a concomitant decrease in phospho-CHK1 [114]. These observations were further supported by studies demonstrating a relative loss of DNA integrity when BCL3 was suppressed following human T cell leukemia virus Type 1 (HTLV1) challenge in T-cells [115] and a role in DNA break repair proposed after profound suppression of the double-strand DNA break repair protein DNA-PK in HeLa cells was observed when BCL3 was suppressed both before and after UVB irradiation [110].

More recently the link between BCL3 and double-strand break repair was reinforced in models of colorectal cancer in which suppression of BCL3 was shown to sensitize to DNA damage-inducing chemotherapy and gamma irradiation [116]. This study initially used siRNA and CRISPR knockdown of BCL3 in human colorectal cancer cell lines to demonstrate sensitization to gamma irradiation via increased double stranded breaks, as indicated by increased phospho-Chk2 and gamma-H2Ax staining foci and a reduction in homologous recombination resulting in reduced RAD51 foci and increased sensitivity to PARP inhibition. This was supported by in vivo studies using BCL3-knockout mice subjected to gamma irradiation or the DNA damage inducing agent cisplatin to demonstrate that intestinal crypts also exhibited increased γH2AX foci and increased cleaved-caspase 3 (CC3) and an increased sensitivity of transgenic CRC mice to chemotherapy in the absence of BCL3. In light of the well characterized increases in BCL3 expression in CRC patient tumours [88, 89] this study provides a rationale for a role of BCL3 in driving resistance to DNA damaging agents in CRC and for the inclusion of BCL3 inhibitors as an adjunct to existing standard of care for CRC patients in the future.

The mechanism by which BCL3 regulated DNA damage repair remains to be elucidated, but most likely involves its protein binding partners within the nucleus, for example the BCL3 binding protein, B3BP, which contains a potential DNA repair motif and exhibits nicking endonuclease activity [117].

### BCL3 and therapy resistance

In addition to its recently proposed role in promoting DNA damage resistance in CRC discussed above [116], BCL3 has been reported to confer resistance to a range of other therapeutic interventions in different tumour contexts. Most of these studies align with the well-established drug-protective effects of NF-kB signaling, yet it should be noted that both WNT and STAT3 signaling pathways are also implicated in cancer drug resistance suggesting a potentially multi-faceted role for BCL3 in driving drug resistance in different tumour contexts [118–120]. We have summarised the studies showing a direct effect of BCL3 on therapy resistance in Table 2.

**Table 2:** Summary of evidence demonstrating direct effects of BCL3 on cancer therapeutic resistance

Cancer Type	Therapy intervention	Bcl3 expression	Ref
CRC cells	DNA damage eg. (Radiotherapy)	↓ Bcl3 expression sensitises to DNA damage	Parker C 2022 [116]
Prostate cancer cells	Staurosporine Etoposide Paclitaxel	↓ Bcl3 expression sensitised apoptosis and cell death	Ahlqvist 2013 [85]
Prostate cancer cells	IL-6	↑ Bcl3 expression protected cells against apoptosis	Ahlqvist 2013 [85]
Glioma	Temozolomide	↑ expression of DcR1 decoy receptor via Bcl3 attenuates TMZ mediated apoptosis	Mansour 2015 [121]
Glioma	Temozolomide	↑ CAII expression via Bcl3 attenuates TMZ mediated apoptosis	Wu 201 8[92]
Gastric Carcinoma cells	Oxoplatin 5-FU Irinotecan	Hypoxia ↑ Bcl3 expression ↓ Bcl3 expression induced sensitivity to chemotherapeutics	Hu 2020 [113]
Breast Cancer cell line	Paclitaxel	↓ Bcl3 expression increased resistance to Paclitaxel	Huo 2018 [40]

Thus the first published evidence supporting a direct role for BCL3 in drug resistance was in 2013 when a study describing elevated expression of BCL3 in prostate cancer (PC) demonstrated enhanced PC cell responses to the chemotherapeutic agents staurosporine, etoposide and paclitaxel when BCL3 was suppressed [85]. This was shown to be, at least partly, through the recruitment of BCL3 to NF-kB binding sites within the promoters of the Helix-Loop-Helix Id proteins, Id1 and Id2. Since then NF-kB/BCL3 signaling has been reported to be responsible for glioma resistance to temozolomide through upregulation of the extrinsic apoptosis inhibitory decoy receptor DcR1 which blocks apoptotic responses via Fas/TRAIL [121], and BCL3-dependent upregulation of the carbonic anhydrase II (CAII) gene via a temozolomide mediated switch between p50/BCL3 and p52/BCL3 complexes at the CAII promoter [92]. The tumour microenvironment likely plays a key role in Bcl3-mediated therapy resistance, for example hypoxia drives BCL3 mediated resistance to oxaliplatin, 5-fluorouracil and irinotecan in gastric cancer cells [113] while interferon gamma, which is often upregulated by chemotherapy [122–124] has been shown in ovarian cancer cells to increase the expression of BCL3 [84] via the Jak/Stat pathway [125]. However, there is a dearth of evidence to date to suggest that BCL3 is upregulated by chemotherapeutic challenge, although a recent study found BCL3 to be a prognostic marker of temozolomide (but not radiotherapy) resistance raising the intriguing question of Bcl3’s role in de novo treatment resistance [92].

BCL3’s effects on chemosensitivity in breast cancer cells has not been explored in depth, yet in contrast to the studies demonstrating BCL3 as a biomarker of poor prognosis in breast cancer (see Table 1), one study has demonstrated a chemo-sensitizing role for BCL3 in a single cell line (MDA-MB-468) representing the BL1 subtype of triple-negative breast cancer. In contrast to all other TNBC subtypes where BCL3 predicts poor overall survival, this subtype alone is associated with improved outcomes [40] and increased resistance to paclitaxel when BCL3 was suppressed. The underlying mechanism for this effect was not elaborated but was associated with increased BCL2 levels.

### BCL3 and immune evasion in cancer

The re-activation of the immune compartment within the tumour microenvironment to promote cell-mediated tumour cell killing shows great promise as a therapeutic strategy and there is intense activity in the pre-clinical and clinical settings to determine ways to enhance tumour surveillance and to further promote immune therapy approaches [126].

One area of interest is in suppressing the inhibitory checkpoints expressed by tumour cells to prevent the activation of cytotoxic T-cells within the tumour microenvironment. One such checkpoint protein, PDL-1, has recently been shown to be under the transcriptional control of BCL3 in ovarian cancer cells [84, 127], suggesting that inhibition of BCL3 may promote immune responses in this cancer type. The mechanism of action was identified in OVCAR3 and SKOV3 cells as a BCL3 mediated recruitment of lys314/315 acetylated p65 NFkB to the PD-L1 promoter.

An additional strategy to enhance immune responses in the tumour microenvironment is to promote the recruitment of immune cells to the tumour site, turning so-called ‘cold’ tumours into immune ‘hot’ tumours that would then have the potential to initiate an activated immune response to the tumour cells.

The induction of IL8 by BCL3 in ovarian cancer cells [125] highlights one presumptive mechanism of BCL3-cytokine driven immune evasion in solid tumours via the recruitment of tumour-associated neutrophils [128]. Moreover, in describing a novel BCL3 inhibitor with anti-cancer properties in models of breast cancer [111], we have reported on the transcriptional responses of a panel of key NFkB-responsive genes to BCL3 inhibition mediated both by siRNA and pharmacologically. These genes included a number of cytokines that have critical roles in the recruitment and activation of immune cells within the tumour microenvironment (Fig. 4). Suppression of BCL3 led to the upregulation of TNF-alpha and IL-6 and the downregulation of IL-10 in MDA-MB-231 triple-negative breast cancer cells [111], all of which would theoretically stimulate the recruitment and activation of immune cells within the tumour microenvironment [129]. Moreover BCL3 has been implicated in TNF-alpha mediated signaling via stabilisation of RIP1 through CYLD ubiquitination in non-cancer cells suggesting that it may also have a role in TNF-alpha responses in the local microenvironment [130]. Together with the potential suppression of PD-L1, this raises the possibility of a dual effector role for BCL3 inhibition in an appropriate tumour microenvironment. Future studies will elucidate the potential for this immune axis in specific tumour contexts.

### BCL3 and cancer stemness

The concept of cancer stemness is based on the experimental evidence that only a small subset of cells within a tumour has capacity at any given time to generate new tumours and that the bulk of the tumour cell population has limited potency. The consequence of this so-called “cancer stem cell hypothesis” for cancer therapy is that it is only a small subset of malignant cells that are responsible for metastatic seeding and disease relapse following therapy and it is this sub-population (which exhibits drug resistance properties) that should be targeted to improve patient outcomes [131, 132].

Intervention on cancer stem cells impacts on a number of the characteristic hallmarks already outlined above and thus evidence of BCL3’s role in driving stemness in cancers likely also overlaps with key properties of metastasis, drug resistance and cell survival.

Clonogenicity, here defined as the ability for single cancer cells to generate a new colony of cells in vitro (e.g. colony formation assay) has been shown to be inhibited following suppression of BCL3 in a number of tumour types, including gastric, colorectal, breast, cervical and ovarian [13, 45, 113, 116]. Whilst a useful measure of multipotentiality of tumour cells in vitro, more rigorous assays for cancer ‘stemness’ include the tumoursphere assay (eg. clonal expansion of suspension organoids in serum deprived media) and the ‘gold-standard’ transplantation of limiting dilutions of tumour cells into recipient mice often used alongside surrogate cell surface markers of stemness. Studies of BCL3 using one or more of these cancer stem cell assays are less common, nonetheless two studies using models of CRC have demonstrated a role for BCL3 in promoting stemness in tumour cells [13, 56]. WNT signaling plays a predominant role in CRC and is a known key signaling pathway in cancer stem cell self-renewal. Unsurprisingly then, when BCL3 was shown in 2019 to enhance β-catenin signaling in a panel of CRC cell lines, that suppression of BCL3 was found to reduce cancer stem cell activity within the tumour populations, as demonstrated by tumoursphere assay and stem cell markers Lgr-5 and Bmi expression [13]. Subsequently this was confirmed in an independent study which provided further in vivo evidence of stemness in a limiting dilution xenograft experiment using CRC cell lines, additional surrogate markers (CD133/CD44/SOX2) and furthermore demonstrating the mechanism of action of this effect mediated through the acetylation of β-catenin at lysine 49 [56].

Based on the definition of CSCs role in tissue of origin and clonal expansion of solid tumours and their role in colonization of tumour cells during metastasis, the therapeutic effect of BCL3 inhibition on cancer stemness has also been described in breast cancer models [111, 133]). Thus, using a stochastic carcinogen-induced mammary tumour model in BCL3-knockout mice, BCL3 was shown to promote the formation of mammary adenocarcinomas through elevated NF-κB leading to the maturation of luminal progenitors [133]. Furthermore, pharmacological suppression of BCL3 systemically in mice inhibited both the experimental metastatic seeding of human triple negative breast cancer (MDA-MB-231) cells and the spontaneous metastasis of syngeneic mammary 4 T1.2 cells in vivo [111].

The likelihood that BCL3 plays a more universal role in regulating cancer cell stemness in other tumour types is supported by the evidence that BCL3 is also permissive for stemness in the non-tumour setting. BCL3 has been demonstrated to link LIF-STAT3 with Oct4 in naive pluripotent stem cells and has been shown to be required for embryonic stem cell renewal through downregulation of Nanog [55, 134]. However, one report describes an inverse relationship between BCL3 and cancer stemness, in which BCL3 appears to reduce the CSC compartment within pancreatic tumours [135], which highlights the heterogeneous nature of CSCs originating from different tissues. Future studies are likely to determine the full extent of BCL3’s role in cancer stemness across different tumour types.

### Opposing roles of BCL3 in cancer

Despite the overwhelming evidence supporting BCL3’s role as an oncogene and driver of disease progression, there have been two reports describing a protective role for BCL3 in specific cancer contexts [40, 135]. One study reported BCL3 as prognostic for improved survival in a specific subtype of triple negative breast cancer patients and in a representative cell line, where BCL3 was shown to improve responses to paclitaxel, while still displaying its conventional oncogenic role in promoting proliferation. In the other study BCL3 was shown to have a tumour suppressive role in pancreatic cancer, reducing the cancer stem cell compartment in these tumours [135]. These studies emphasise the importance of patient stratification and tumour context when considering the therapeutic potential of new cancer targets.

Additionally, in a mouse model of colitis-associated colorectal cancer whereby tumours are initiated through the addition of azoxymethane/dextran-sodium sulphate, BCL3 was shown to have a protective effect [136]. Conditional knockdown of BCL3 in this mouse model resulted in significantly more polyp formation than wild type controls. The combined knockdown of BCL3 and TNFα blocked this increase, suggesting the ‘tumour initiating phenotype” of suppressed BCL3 was mediated by TNFα.

## Targeting BCL3 in cancer

The weight of evidence determines that BCL3 is a multifaceted regulator of key cancer pathways associated with many of the hallmarks of cancer. Recent evidence in the past 10 years now indicates that beyond its canonical role as a co-factor for NFkB signaling, the BCL3 protein modulates cancer progression and therapy resistance through distinct and novel mechanisms involving protein modifications and interactions with alternative oncogenic protagonists including c-Myc, WNT/β-catenin and STAT3.

This places BCL3 as a peripheral modifier of a network of transcription factor pathways that influence oncogenesis and transition to metastasis in different tissue contexts and identifies BCL3 as a potential therapeutic target. Here, we summarise the attributes of BCL3 that support its candidacy as a promising therapeutic target.

### BCL3 loss is well tolerated

BCL3 knockout mice are viable with relatively mild defects in antigen-specific B- and T-cell responses compared to comparable knockouts of the NF-kB subunits [34, 36, 137]. Thus, targeting BCL3 in adult tissues would be predicted to have modest side effects while impacting on aberrant pathways in cancer, as evidenced by the studies described above where BCL3 suppression impacts on cancer outcomes. We propose therefore that suppression of BCL3 limits and modifies the related oncogenic pathways to an extent that suppresses malignancy but is otherwise tolerated.

### BCL3 modulates multiple pathways

Targeting a protein that has a role in a very specific pathway has the advantage of specificity but potentially lacks the ability to influence compensatory mechanisms and redundancy that leads to evasion of treatment. As BCL3 modifies multiple signaling pathways, yet is well tolerated, it has the potential to disrupt both the oncogenic drivers, and evading mechanisms inherent in cancer treatment responses.

### BCL3 promotes both primary tumour growth and secondary metastasis

With evidenced roles in promoting primary tumour growth in a number of tumour types, while also driving metastatic spread (the primary cause of cancer patient deaths), suppressing BCL3 would seem to have dual benefits in providing clinically measurable beneficial outcomes on primary tumour growth while also potentially providing significant benefits for long term survival. It is recognized that a clinically relevant anti-metastatic agent would have appreciable impact on patient outcomes, yet the current clinical trials landscape does not accommodate ‘pure’ anti-metastatics [138]. BCL3 meets the requirements of a therapeutic target that addresses both primary tumour growth and metastasis.

### Lowering BCL3 levels is sufficient to impact on cancer progression

This review has focused on the studies demonstrating direct effects of suppressing BCL3 activity on cancer progression and the majority of these have used BCL3-gene knockdown or knockout as the modality of BCL3 inhibition. With the advent of new RNA delivery modalities and recent successes of RNA-based anti-virals, RNA-based therapeutics for cancer, including miRNAs, have garnered renewed interest [139]. BCL3 would be an attractive candidate for such an approach as the body of evidence (above) shows that a reduction in BCL3 expression has anti-cancer properties in a variety of contexts. This is supported by studies reporting specific micro-RNAs that target BCL3 to ameliorate cancer hallmarks. These include mir125b in ovarian cancer [140], mir627-5p in hepatocellular carcinoma [141, 142], mir216a-3p in choliangiocarcinoma [143] and miR1343-3p in non-small cell lung cancer [144].

### Targeting BCL3 protein complexes

BCL3 mediates its effects through protein:protein interactions and disruption of these protein complexes have been shown to exhibit anti-cancer properties [27, 111]. The best-defined molecular interface on BCL3 is its ankyrin repeat domain, as this was identified as the region required for p50/p52 interaction [23, 24]. Modelling of this interface identified a unique pocket in the carboxy-terminal region of the ankyrin repeat that could be suitable for drug-like molecules binding, and subsequent in silico screening in this region identified a novel small-molecule capable of inhibiting BCL3:p50 binding [111]. This small-molecule inhibitor exhibited marked anti-tumour and anti-metastatic properties in mouse models of breast cancer [111]. The BCL3 domains required for other protein interactions are less well defined. The use of deletion mutations of BCL3 suggest that the interaction with β-catenin requires the P-rich amino-terminal region of BCL3 bordering the ankyrin domain, however it remains to be determined whether disruption of the ankyrin repeat itself impacts on β-catenin binding [56]. What is clear is that recruitment of BCL3 either modulates the acetylation status of the bound proteins (for example NF-kB (p50/p52) and β-catenin) and recruitment of histone deacetylases to these complexes [22, 56, 99, 145] or it regulates ubiquitination and degradation of the binding partners (for example SMAD3 and c-Myc) [11, 14, 58] to regulate their activity which impacts on oncogenicity.

An alternative approach to the disruption of BCL3 protein complexes is to target the interacting protein directly. One reported example of this is the description of a small molecule inhibitor of PIRIN, which disrupts Pirin:BCL3 binding, which leads to the inhibition of melanoma cell line migration [27].

These initial proof of concept studies for the use of small molecule protein:protein inhibitors (PPIs) of BCL3 indicates the significant potential for these novel classes of BCL3 inhibitor as anti-cancer agents. Our improved understanding of these protein complexes and the downstream consequences of their disruption will further contribute to the appropriate application of these inhibitors in the clinical setting.

### Post-translational modification of BCL3

BCL3 is a heavily modified protein which regulates its stability, subcellular localisation and activity and impacts on its oncogenicity [29, 31, 52, 100, 146, 147]. Whether these modifications which include phosphorylation and ubiquitination (reviewed by [16, 17]) could be harnessed directly or through targeting of BCL3 upstream modifiers, as suggested for the cancer related deubiquitination enzyme CYLD [148], remains to be determined. Currently no specific BCL3 protein modification modalities have been described, and any upstream targets such as CYLD are likely to exhibit effects on other signaling proteins raising the potential for off-target toxicities.

## Summary

BCL3 has been implicated in most of the hallmarks of cancer and as a modifier of disease progression and drug resistance impacts directly on key areas of clinical unmet need.

Targeting BCL3 is challenging, not least because of the multiplicity of its actions on transcriptional pathways and contradictory roles in some tumour models. Yet its direct role as an independent risk factor in some major cancer subtypes, such as colorectal and breast, and the evidence that it is well tolerated when inhibited in animal models highlights it as a promising therapeutic target in these clinical settings. Targeting the BCL3 interactome directly already demonstrates great promise as a therapeutic strategy [111]. With well evidenced roles in both primary tumour viability and progression to metastasis targeting BCL3 has potential to impact on long-term survival of patients in a number of cancer sub-types.

## Data Availability

Not applicable.

## Abbreviations

BCL3: B-cell lymphoma 3
WNT: Wingless-related integration site
NF-kappaB: Nuclear Factor Kappa B
STAT3: Signal transducer and activator of transcription 3
SMAD3: Mothers against decapentaplegic homolog 3
C-Myc: myelocytomatosis
TGFb: transforming growth factor beta
CYLD: cylindromatosis
